# Laparoscopically assisted percutaneous endoscopic gastrostomy performed for remnant stomach in patient with amyotrophic lateral sclerosis: a case report

**DOI:** 10.1186/s40792-023-01683-y

**Published:** 2023-06-07

**Authors:** Yutaro Ohgaki, Yuji Ishibashi, Fumihiko Hatao, Ryuichiro Furuta, Noriyuki Saito, Rie Inayoshi, Yasuhiro Morita

**Affiliations:** 1 Department of Surgery, Tokyo Metropolitan Tama Medical Center, 2-8-29 Musashidai, Fuchu, Tokyo 183-8524 Japan; 2 Department of Anesthesiology, Tokyo Metropolitan Tama Medical Center, 2-8-29 Musashidai, Fuchu, Tokyo 183-8524 Japan

**Keywords:** Gastrostomy, Laparoscopic surgery, Amyotrophic lateral sclerosis

## Abstract

**Background:**

Although percutaneous endoscopic gastrostomy (PEG) offers better access to the gastrointestinal system, in patients with previous abdominal surgery, PEG can be unsuccessful. Laparoscopically assisted percutaneous endoscopic gastrostomy (LAPEG) is indicated for such patients. However, patients with amyotrophic lateral sclerosis (ALS) may be more susceptible to anesthesia-related complications than other patients, requiring the indications for LAPEG, along with perioperative management, to be considered carefully.

**Case presentation:**

A 70-year-old, male patient with ALS was referred to our hospital for a gastrostomy for progressive dysphagia. He had undergone an open distal gastrectomy for gastric ulcer perforation in his twenties. Upper gastrointestinal endoscopy denied the transillumination sign and focal finger invagination. Because the risk of respiratory complications caused by general anesthesia was not considered serious, the decision was made to perform a LAPEG. Under careful, intraoperative airway management and neuromuscular monitoring, adhesiolysis was performed to increase mobility of the remnant stomach. A gastrostomy tube was inserted through the abdominal wall and into the remnant stomach under laparoscopic and endoscopic guidance. The patient was discharged in stable condition on postoperative day 3 without any respiratory complications.

**Conclusions:**

LAPEG was able to be performed in a patient with ALS with a previous gastrectomy. A perioperative team comprised of neurologists, endoscopists, surgeons, anesthesiologists, and nurses who are fully conversant with ALS must be assembled to deal with potentially complex medical issues related to the procedure and anesthetic and perioperative management.

## Background

Amyotrophic lateral sclerosis (ALS) is a neurodegenerative disease characterized by progressive muscle weakness caused by motor neuron dysfunction [1]. In many cases, dysphagia is the initial symptom and constitutes one of the most serious complications [2]. When dysphagia becomes progressive, placement of a gastrostomy tube should be considered. Although percutaneous endoscopic gastrostomy (PEG) offers better access to the gastrointestinal system than other surgical methods, in patients with previous abdominal surgery, especially upper abdominal surgery, PEG placement can be unsuccessful, and surgical placement of a gastrostomy tube may be required. Several, recent studies of laparoscopically assisted percutaneous endoscopic gastrostomy (LAPEG) have reported good results [3–7]. However, patients with ALS may be more susceptible to anesthesia-related complications than other patients [8], requiring the indications for LAPEG as well as perioperative management to be considered carefully. Herein, we report a case of LAPEG for remnant stomach in a patient with ALS.

## Case presentation

**Fig. 1:**
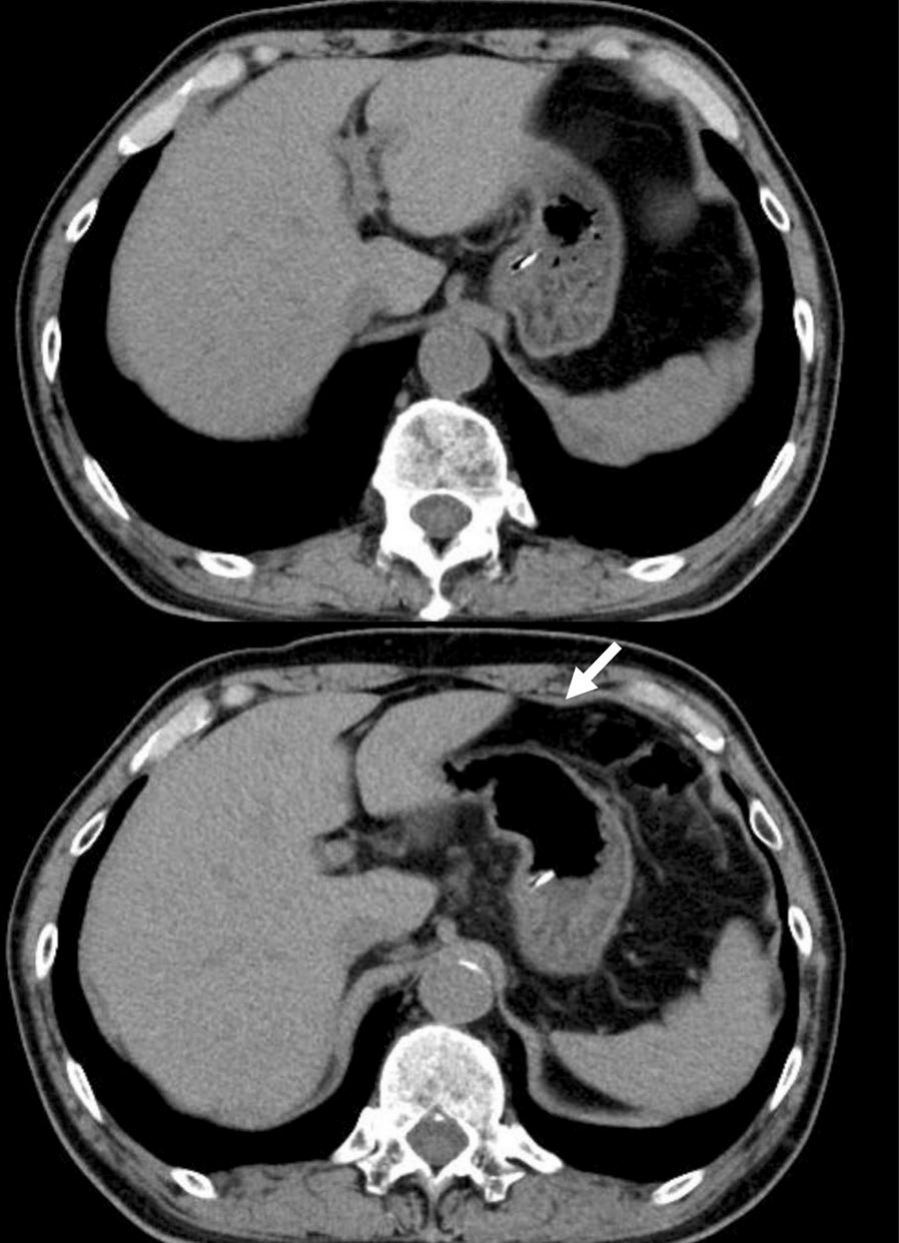
Computed tomography revealed the left lateral segment of the liver and transverse colon (arrow) between the remnant stomach and abdominal wall

**Fig. 2:**
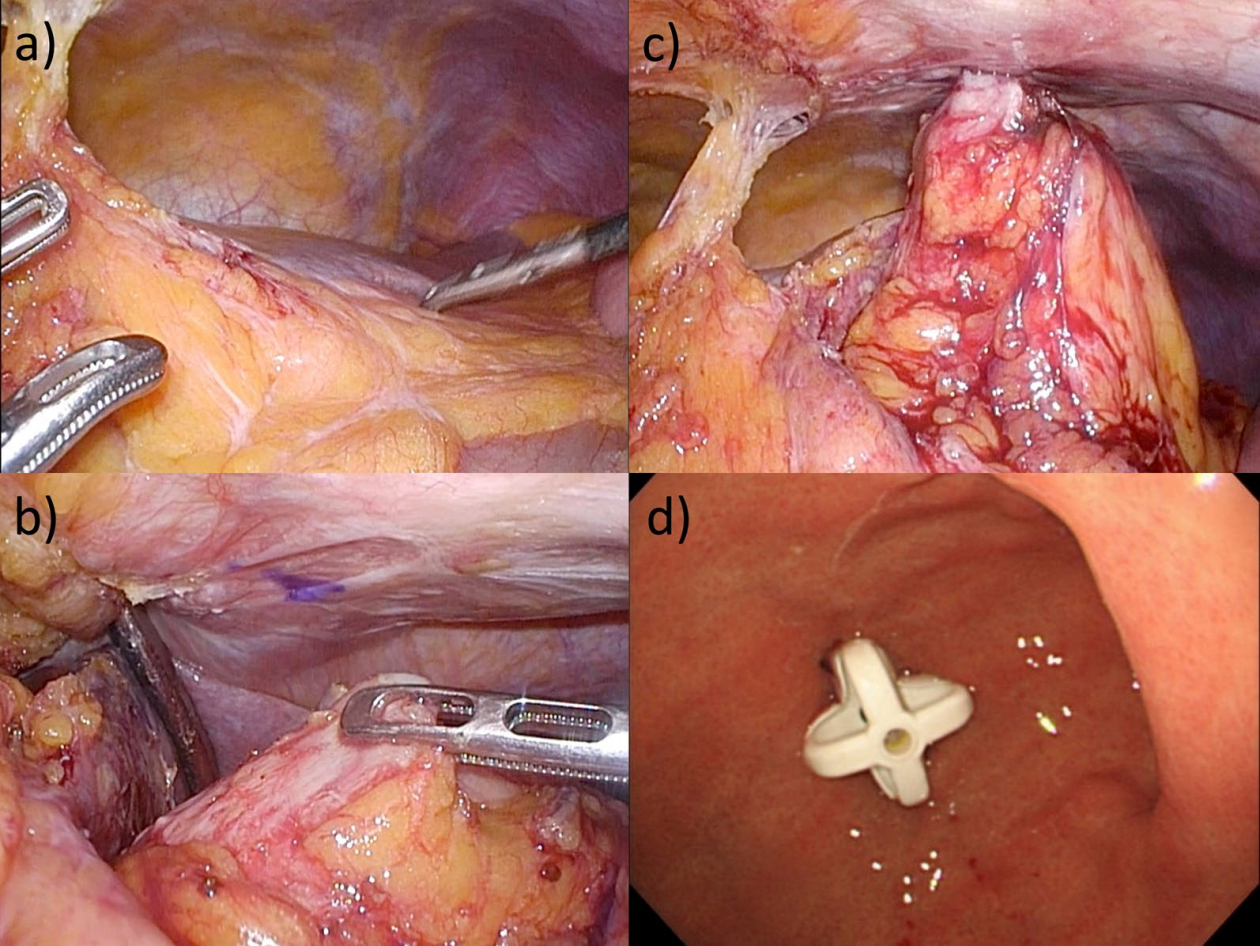
Intraoperative findings. **a** Adhesiolysis was performed to increase mobility of the remnant stomach. **b**, **c** Gastric wall and the abdominal wall were brought closer by lowering the pneumoperitoneum pressure and were fixed with a gastropexy device. **d** Placement of the gastrostomy was confirmed by laparoscopically and endoscopically

A 70-year-old, male patient had received a diagnosis of ALS 4 years previously and was recently referred to our hospital for a gastrostomy for progressive dysphagia. He had undergone an open distal gastrectomy and Billroth-I reconstruction for gastric ulcer perforation in his twenties. Upper gastrointestinal endoscopy denied the transillumination sign and focal finger invagination. Computed tomography revealed the left lateral segment of the liver and transverse colon between the remnant stomach and abdominal wall (Fig. 1). An endoscopic or radiologic gastrostomy was considered difficult to perform. The patient’s respiratory function demonstrated vital capacity (VC) 3.36 L, %VC 95.5%, forced vital capacity (FVC) 3.33 L, and %FVC 94.6%. He had a score of 28 on the amyotrophic lateral sclerosis functional rating scale-revised (ALSFRS-R). The patient denied respiratory complaints and had no history of pulmonary disease. Because the risk of respiratory complications caused by general anesthesia was not considered serious, the decision was made to perform a LAPEG, which is less invasive than a laparotomy with gastrostomy. Before tracheal intubation, propofol 100 mg and rocuronium 20 mg were administered. Anesthesia was maintained with sevoflurane in 50% oxygen with continuous infusion of remifentanil under careful neuromuscular monitoring. In total, 34 mg of rocuronium was used intraoperatively as a muscle relaxant. An epidural catheter was inserted, and an epidural infusion of ropivacaine 0.2% was given throughout the surgery for analgesia. The remnant stomach was located below the liver and adhered to the liver, omentum, and transverse mesocolon. Adhesiolysis was performed to increase mobility of the remnant stomach. Endoscopy was then performed, and the remnant stomach was insufflated. The gastrostomy site was confirmed via laparoscopy and endoscopy. The gastric wall and the abdominal wall were brought closer by lowering the abdominal pressure (10–5 mmHg) and were fixed in place using a gastropexy device. A gastrostomy tube (EndoViveTM Button II 20Fr 4.5 cm; Boston Scientific Japan, Tokyo, Japan) was inserted via the introducer method through the abdominal wall and into the remnant stomach, and the placement of the gastrostomy was confirmed laparoscopically and endoscopically (Fig. 2). The total operative time was 129 min, and the blood loss was 2 mL. The patient was extubated in the operating theater, then moved to the intensive care unit (ICU) for observation of the vital signs and respiratory status. After ICU admission, his blood gas analysis (BGA) under 5 L/min of oxygen showed PaO_2_ 182.0 mmHg and PaCO_2_ 39.5 mmHg. His respiratory status was stable, and he had no respiratory fatigue. On postoperative day (POD) 1, he was moved to a recovery ward after his BGA under room air showed PaO_2_ 85.0 mmHg and PaCO_2_ 40.3 mmHg and his respiratory status had stabilized. Feeding via PEG tube was begun on POD 2. The patient was discharged home in stable condition on POD 3 without any respiratory complications.

## Discussion

Enteral nutrition is necessary in patients for whom oral nutrition is insufficient or impossible. Nasogastric tube insertion can be readily performed and is useful in the short term as a form of enteral feeding but can have lower feeding efficacy as well as the risk of potential complications, such as irritation, ulceration, bleeding, esophageal reflux, and aspiration pneumonia. Patients sometimes also report discomfort with this procedure [9]. In cases where long-term enteral nutrition is required, placement of a gastrostomy tube may be considered. PEG was introduced by Gauderer et al. as an alternative to the laparotomy gastrostomy [10]. Besides its well-known advantages over parenteral nutrition, PEG offers superior access to the gastrointestinal system than other surgical methods [9]. The procedure is easy to perform, less invasive, and has been widely used with good outcomes [11]. However, it is contraindicated for patient with severe ascites, peritonitis, serious coagulation disorder or gastrointestinal obstruction [9, 12]. Although the gastrostomy tube can be inserted in patients with previous abdominal surgery involving the stomach after confirming a safe tract with no interposed organs, PEG insertion is difficult to perform [9, 13]. An endoscopic or radiologic gastrostomy may be considered as an alternative, although these methods are sometimes technically demanding and require specialized equipment and experienced personnel [14].

If an endoscopic or radiologic gastrostomy is not possible, LAPEG should be considered as a minimally invasive alternative to the open gastrostomy. LAPEG, which was described for the first time by Raaf et al. [15], eliminates the risk of blind injury to the viscera and allows the optimal site for gastrostomy placement in both the stomach and abdominal wall to be determined. LAPEG also enables the stomach to be pulled into a normal position and other organs overlying the stomach to be avoided under direct observation via laparoscopy. Barkmeire reported a higher success rate for laparoscopically assisted techniques (100%) than for conventional PEG (84%), although the procedural and postprocedural complication rates were similar [16]. Some studies have reported successful LAPEG in patients with previous abdominal surgery of any kind [3, 6]. However, LAPEG can be highly invasive in cases where adhesiolysis of the remnant stomach is difficult or poses a high risk of bleeding or damage to other organs or the operative time is significantly prolonged. In such cases, a laparoscopically assisted jejunostomy, open gastrostomy or open jejunostomy should be considered as an alternative. In the present case, LAPEG was planned, because the patient opted for a gastrostomy rather than nasogastric tube insertion or a jejunostomy, because tube and nutritional management was easier with a gastrostomy than with other methods. The adhesion of the remnant stomach and other organs was not severe, allowing adhesiolysis to be performed easily and the LAPEG to be performed less invasively.

In patients with ALS, perioperative management for preventing respiratory complications is extremely important, because critical, postoperative, respiratory complications can lead to decreased quality of life. In preoperative management, the results of serial pulmonary function tests and ALSFRS-R assessment during the preoperative period should aim to evaluate the functional status of these patients and determine whether they may require postoperative mechanical ventilation [17]. To minimize the risk of respiratory complications in patients with ALS, the current guidelines recommend that the patients undergo PEG before their VC decreases to 50% of the normal value [18]. For intraoperative management, careful monitoring and appropriate drug selection are necessary, especially in surgery requiring general anesthesia and muscle relaxants. In patients with ALS, an abnormal response to a muscle relaxant can lead to respiratory depression, and depolarizing neuromuscular blocking agents (succinylcholine) have the potential to induce hyperkalemia [17]. In the present case, surgery was performed under general anesthesia using a low-dose, non-depolarizing neuromuscular blocking agent (rocuronium) and epidural anesthesia with careful airway management and intraoperative neuromuscular monitoring. In postoperative management, aggressive physiotherapy is recommended to restore pulmonary function. Although the present patient was an exception, mechanical ventilation should be performed in patients with respiratory muscle weakness. Noninvasive ventilation (NIV) reduces respiratory fatigue, promoting CO_2_ washout and better oxygenation. Some studies have reported that NIV is effective in ALS patients after elective surgery under general anesthesia as means of safely preventing postoperative respiratory failure [19, 20].

There are no previous studies describing the details of LAPEG in patients with ALS, and the safety of laparoscopic surgery in these patients is still unclear. Diaphragmatic pacing, used to stimulate the diaphragm to prevent atrophy and prolong life by preventing pulmonary complications, and laparoscopic diaphragmatic pacing have sometimes been performed in patients with ALS. A multicentric study of these methods demonstrated that laparoscopic diaphragm surgery can be performed safely in patients with ALS [21]. It is possible that laparoscopic surgery, including LAPEG, can also be performed safely in patients with ALS.

## Conclusion

In the present case, LAPEG was able to be performed in a patient with ALS with a history of gastrectomy. A perioperative team comprising neurologists, endoscopists, surgeons, anesthesiologists, and nurses who are fully conversant with ALS must be assembled to deal with the potentially complex medical conditions related to the procedure as well as to anesthetic and perioperative management.

## Data Availability

All the data generated or analyzed during this study are included in this article.

## Abbreviations

ALS: Amyotrophic lateral sclerosis
PEG: Percutaneous endoscopic gastrostomy
LAPEG: Laparoscopically assisted percutaneous endoscopic gastrostomy
VC: Vital capacity
FVC: Forced vital capacity
ALSFRS-R: Amyotrophic lateral sclerosis functional rating scale-revised
ICU: Intensive care unit
BGA: Blood gas analysis
POD: Postoperative day
NIV: Noninvasive ventilation
